# Perception of patients with temporomandibular disorders of the effects of botulinum toxin application: a retrospective cross-sectional observational study

**DOI:** 10.1007/s00784-026-07070-9

**Published:** 2026-08-08

**Authors:** Franco Ignáccio Mallaguti, José Geraldo Mallaguti, Otacilio de Paula Rodrigues Júnior, Brenda Moliterno de Carvalho, Analia Gabriella Borges Ferraz Facury, Ana Paula Ayres Oliveira

**Affiliations:** 1 Dental Clinic, Instituto de Tecnologias Odontológicas Avançadas Malaguti, Uberaba, MG Brazil; 2 Medical school, Universidade de Uberaba, Uberaba, MG Brazil; 3 Restorative Dentistry, Universidade de Uberaba, Uberaba, MG Brazil; 4 Universidade de Uberaba, 1801 Nenê Sabino Ave., Universitário, Uberaba, MG, Zip 38055-500 Brazil

**Keywords:** Botulinum A Toxin, Temporomandibular Disorder, Clinical study, Questionnaire, Bruxism

## Abstract

**Objective:**

To evaluate patient-reported changes in orofacial and masticatory symptoms after BoNT-A application in individuals presenting bruxism-related complaints.

**Materials and Methods:**

A retrospective cross-sectional observacional study was conducted with 61 adult participants (47 women, 14 men) who underwent BoNT-A treatment for bruxism. An online questionnaire, assessed masticatory and orofacial symptoms before and after treatment, using a five-point frequency scale. Data were analyzed with descriptive statistics, paired t-tests, and Spearman’s correlation (p < 0.05). This observational study was reported in accordance with the STROBE guidelines.

**Results:**

A statistically significant reduction in total symptom scores was observed after treatment (*p* < 0.05), with a mean decrease from 32.4 ± 11.5 (before) to 15.6 ± 6.59 (after), representing an overall reduction of approximately 52%. No significant differences in symptom improvement were observed between sexes or in relation to age. Although an overall difference among professional categories was identified, post hoc analysis did not show significant pairwise differences between groups.

**Conclusion:**

Individuals with bruxism-related complaints reported improvement in pain-related and masticatory symptoms after BoNT-A application. BoNT-A may represent a supportive approach for managing orofacial and muscle-related complaints; however, further studies with standardized diagnostic criteria and objective outcome measures are needed to clarify its long-term clinical effects.

**Clinical Relevance:**

BoNT-A application may contribute to the management of pain-related and masticatory complaints reported by individuals with bruxism-related symptoms, supporting its potential use as an adjunctive approach in selected clinical situations.

**Graphical Abstract:**

**Figure:**
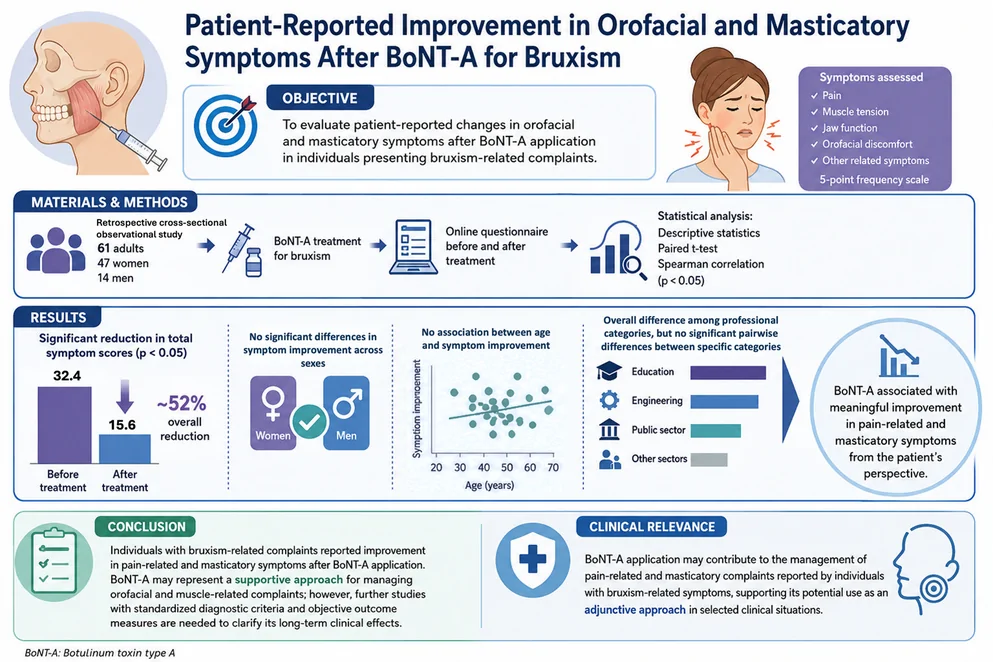

## Introduction

Temporomandibular disorders (TMD) have been defined by the American Association for Dental Research (AADR) as “a set of musculoskeletal and neuromuscular conditions that affect the temporomandibular joints (TMJ), masticatory muscles, and all associated tissues” [1]. Approximately 44% of the population is affected, yet only a quarter seek professional help [2]. TMD may result in orofacial pain, jaw discomfort, and reduced quality of life. The etiology of TMD is complex and multifactorial, with factors such as nocturnal bruxism, jaw clenching, stress, anxiety, and depression frequently associated with symptom development and exacerbation [2, 3].

Among parafunctional jaw activities, bruxism is one of the most frequently reported conditions. Previous studies have estimated a prevalence of approximately 18.6% in adults, with sleep bruxism affecting approximately 15.9% and awake bruxism approximately 23.8% of the population [4]. Bruxism is currently defined as a repetitive jaw-muscle activity characterized by clenching, grinding, bracing, or thrusting of the mandible, and may occur during wakefulness or sleep [1]. Although the relationship between bruxism and temporomandibular disorders remains complex and not fully established, some individuals with bruxism-related behaviors may report symptoms such as muscle fatigue, jaw discomfort, headaches, and dental wear [4, 5].

Management of temporomandibular and bruxism-related symptoms is generally based on a multifactorial and individualized approach aimed at reducing pain, improving masticatory function, and minimizing the impact of symptoms on daily life. Conservative approaches are usually considered the first-line option and may include physiotherapy, behavioral counseling, control of parafunctional habits, use of occlusal appliances, pharmacological therapy such as analgesics or muscle relaxants, and psychosocial interventions focused on stress management [1, 3]. More invasive procedures are typically reserved for selected cases with persistent or severe symptoms that do not respond adequately to conservative management.

Botulinum toxin (BoNT) has emerged over the past decades as a potential adjunctive approach for the management of orofacial pain and masticatory muscle complaints. It is a neurotoxin produced by the anaerobic bacterium Clostridium botulinum [2]. In the orofacial region, BoNT is commonly injected into masticatory muscles such as the masseter and temporalis, particularly in patients presenting muscle-related symptoms and parafunctional behaviors [3]. Botulinum toxin type A (BoNT-A) acts primarily by inhibiting acetylcholine release at the neuromuscular junction, leading to temporary reduction in muscle contraction [6]. In addition to its neuromuscular effects, growing evidence suggests that BoNT-A may also exert antinociceptive effects through modulation of peripheral and central nociceptive pathways, including inhibition of inflammatory neuropeptides and excitatory neurotransmitters such as substance P and glutamate [7]. Although the mechanisms and clinical indications of BoNT-A in temporomandibular disorders and bruxism-related symptoms are still under investigation, previous studies have suggested that this approach may contribute to symptom reduction and improvement in patient-reported outcomes [8]. Nevertheless, evidence regarding patient-reported symptom changes after BoNT-A treatment remains limited, particularly in real-world clinical settings, highlighting the need for further investigation.

Within this context, the aim of the present study was to evaluate patient-reported changes in orofacial and masticatory symptoms after BoNT-A application in individuals presenting bruxism-related complaints. The null hypothesis was that no differences would be observed in patient-reported symptoms before and after BoNT-A application.

## Materials and methods

### Study design

Patients treated at a private dental clinic owned by one of the researchers involved and who underwent BoNT-A application therapy were screened. Those who received the procedure due to a diagnosis of bruxism or teeth clenching were selected and invited to participate in the present study. This was a retrospective cross-sectional observacional study evaluating patient-reported symptoms before and after BoNT-A application in individuals with bruxism-related complaints through an online questionnaire, and was approved by Research Ethics Committee (CEP – Brazil), CAAE 77836524.1.0000.5145. This study was conducted following the Strengthening the Reporting of Observational Studies in Epidemiology (STROBE) guidelines.

### Participants

Patients invited to participate in the present study received BoNT-A application treatment at the Malaguti Institute of Advanced Dental Technologies (Uberaba/MG). Medical records and treatment forms of all patients who underwent the procedure were analyzed for data tabulation and screening, allowing for inclusion or exclusion from the invitation to complete the questionnaire. Initially, the estimated number of eligible participants for the study was 107 patients.

- Inclusion criteria: (1) having undergone BoNT-A application; and (2) diagnosis of probable awake and/or sleep bruxism based on self-report associated with clinical findings recorded in the dental charts (e.g., tooth wear, masseter hypertrophy and/or other clinical signs), according to the contemporary diagnostic grading recommendations [9].
- Exclusion criteria: (1) age under 18 years; (2) incomplete clinical records preventing confirmation of the diagnosis or collection of the study variables; and (3) use of an occlusal splint during the study period, refusal to participate, or failure to complete the questionnaire.

Eligible participants were contacted by phone and invited to participate regardless of the time elapsed since BoNT-A application. Those who agreed received the questionnaire electronically after providing informed consent. Participant confidentiality was ensured, and individuals could withdraw from the study at any time without affecting their treatment.

### BoNT-A injection protocol

All participants had previously received BoNT-A application as part of their clinical management for bruxism symptoms. The procedure was performed by an experienced dentist trained in facial neuromuscular injections, following a standardized protocol.

Commercially available BoNT-A (Botox^®^, Allergan Aesthetics, USA) was reconstituted with sterile saline (0.9% NaCl) at a dilution ratio of 1 mL per 100 U. Injections were performed bilaterally under aseptic conditions using insulin syringes.

For each side, a total dose of 25 U was administered, consisting of 20 U into the masseter muscle and 5 U into the temporalis muscle. Masseter injections were performed at two standardized points in the lower third of the muscle belly (10 U per point), while temporalis injections were administered at one standardized point in the anterior portion of the temporalis muscle identified by palpation during voluntary contraction.

Thirty minutes before injection, a 4% lidocaine topical anesthetic cream (Dermomax^®^, Biosintética, São Paulo, SP, Brazil) was applied over the masseter regions. Standard post-procedure recommendations were provided to minimize toxin diffusion and migration to unintended sites.

### Online Questionnaire

To evaluate patient-reported symptoms before and after BoNT-A application, participants completed a single online questionnaire after BoNT-A treatment, retrospectively reporting their symptoms before treatment and their perceived symptoms after treatment. The questionnaire also collected personal data and was specifically developed for the present study to investigate self-reported symptoms associated with masticatory discomfort and daily functional limitations. The questionnaire was created using the Google Forms tool, following the guidelines for conducting research with procedures carried out in a virtual environment, as outlined by the Brazilian National Research Ethics Commission (CONEP, 2021). Table 1 shows the specific questions related to the BoNT-A application and theirpossible answers (scores).

**Table 1:** Specific questions regarding BoNT-A application treatment at the Malaguti application and corresponding response scores

Before TxB application	1	2	3	4	5
Did you have difficulty chewing any food?					
Did you have difficulty opening and closing your mouth?					
Overall, did you feel pain in your mouth?					
Did you feel pain in the jaw (lower part of the mouth)?					
Did you have headaches?					
Did you feel discomfort when eating any food?					
Did you feel pain while speaking?					
Did you have to avoid eating certain foods?					
Did you have to interrupt meals?					
Was your sleep disrupted?					
If you experienced pain and needed to take medication, how often did you use it?					
After TxB application	1	2	3	4	5
Do you have difficulty chewing any food?					
Do you have difficulty opening and closing your mouth?					
Overall, do you feel pain in your mouth?					
Do you feel pain in the jaw (lower part of the mouth)?					
Do you have headaches?					
Do you feel discomfort when eating any food?					
Do you feel pain while speaking?					
Do you have to avoid eating certain foods?					
Do you have to interrupt meals?					
Is your sleep disrupted?					
If you experience pain and need to take medication, how often do you use it?					

Scores: 1 = never; 2 = almost never; 3 = occasionally; 4 = sometimes; 5 = always

The online form was divided into five sections:

- Section 1 : introduction to the study, administrative information (including researcher contact details), and a question about the participant’s age.
- Section 2 : presentation of the Informed Consent Form (ICF) and acceptance or refusal to participate.
- Section 3 : collection of general data (sex, age, occupation, email, nationality, and city of residence).
- Section 4 : questions regarding oral health status before application BoNT-A.
- Section 5 : questions regarding oral health status after application BoNT-A.

Participants completed the questionnaire independently through the online platform. The completed questionnaires were accessed and analyzed only by a member of the research team who was not involved in the participants’ clinical treatment, and all information was handled confidentially to encourage honest and unbiased responses. At the end of Sections 1 and 2, if the participant selects that they are under 18 years old or declines to participate in the study, they were automatically directed to complete the form without proceeding further. All objective questions in the form were marked as required (Google Forms configuration), so participants were not able to proceed if they are under 18, unaware of the study, or have not read and agreed to the ICF. However, for all other questions, there were an option “prefer not to answer”. This option ensured participants the possibility of skip any question with no justification needed.

At the end of the form submission, participants were automatically received by email a copy of the completed form along with the ICF.

### Statistical analysis

The information collected through the questionnaire was tabulated and stored in a restricted, controlled, and secure electronic environment managed by the research team. For data analysis, the possible answers—never, almost never, occasionally, sometimes, and always— were converted into numerical scores ranging from 1 to 5, respectively, so that higher scores indicated greater frequency or intensity of symptoms.

Professional data were categorized into eight groups according to area of activity (health, education/psychopedagogy, administration/public sector/law, business/entrepreneurship, engineering/agronomy, retirees/homemakers/unemployed, students, and general services/other).

No a priori sample size calculation was performed, as the study included all consecutive eligible patients treated within the study period. A posteriori sensitivity analysis indicated that with *n* = 61 and α = 0.05, the study had 80% power to detect within-subject effects of approximately dₓ = 0.36 or greater.

Data were analyzed using Jamovi software (version 2.6.22.0). Descriptive statistics were initially performed to characterize the sample and study variables. The Shapiro–Wilk test was applied to verify data normality and define the appropriate statistical tests. Because the paired differences between pre- and post-treatment questionnaire scores met the assumption of normality (*p* = 0.201), comparisons were performed using the paired Student’s t-test, adopting a significance level of 5% (*p* < 0.05).

Given the ordinal nature of the questionnaire scores and the non-normal distribution of some variables, Spearman’s correlation test was performed to assess the association between age and symptom improvement. Comparisons in symptom improvement between sexes were performed using the Mann–Whitney U test, while comparisons in symptom improvement among professional groups were performed using the Kruskal–Wallis test. The Spearman’s rho (ρ) coefficient was interpreted based on strength and direction: values near + 1 indicate a strong direct correlation, values near − 1 indicate a strong inverse correlation, and values close to 0 indicate weak or no correlation.

## Results

A total of 61 participants were included in the study, comprising 47 women and 14 men. The mean age was similar between women (42.9 years) and men (42.5 years). A statistically significant reduction in bruxism symptom questionnaire scores was observed after BoNT-A treatment (Paired Samples T-Test, *p* < 0.001) (Table 2). Overall scores decreased from 1,975 before treatment to 950 after treatment, corresponding to an overall reduction of approximately 52%.

**Table 2:** Participant characteristics and bruxism symptom questionnaire scores before and after BoNT-A treatment

Variable	Total (*n* = 61)
Age, mean ± SD	42.8 ± 14.9
Age, median (min–max)	41 (18–74)
Symptom score before	32.4 ± 11.5
Symptom score after	15.6 ± 6.59
Paired Samples T-Test	*p* < 0.001

When questionnaire scores were analyzed according to professional category (Table 3), reductions were observed in nearly all groups. The greatest descriptive reductions were found among participants working in education/psychopedagogy (− 71%), engineering/agronomy (− 65%), and administration/public sector/law (− 63%).

**Table 3:** Descriptive changes in bruxism symptom questionnaire scores according to professional category

Professions	*N*	Times	Scores	Differences between scores
Health professionals	27	Before	740	− 42%
		After	432	
Education/psych pedagogy	5	Before	214	− 71%
		After	62	
Business/entrepreneurship	10	Before	313	− 52%
		After	151	
Administration/public sector/law	6	Before	221	− 63%
		After	82	
Engineering/agronomy	6	Before	130	− 65%
		After	46	
Retirees / Homemakers / Unemployed	6	Before	243	− 56%
		After	107	
Students	2	Before	79	− 67%
		After	26	
General Services/Other	1	Before	35	+ 3%
		After	36	

Analysis of demographic variables and symptom improvement (Table 4) showed no significant association between age and symptom improvement (Spearman’s ρ = 0.060, *p* = 0.647), and no significant difference in symptom improvement between women and men (Mann–Whitney U = 326, *p* = 0.966). Symptom improvement differed significantly among professional groups (Kruskal–Wallis χ² = 16.6, *p* = 0.020); however, post hoc Dwass–Steel–Critchlow–Fligner (DSCF) pairwise comparisons did not reveal significant differences between specific professional categories.

**Table 4:** Association between demographic variables and symptom improvement following BoNT-A treatment

Variable	Statistical test	Statistic	*p*-value
Sex	Mann–Whitney U	U = 326	0.966
Age	Spearman’s rho	ρ = 0.060	0.647
Profession	Kruskal–Wallis	χ² = 16.6	0.020*

Statistical significance was set at *p* < 0.05. For professional groups, significant overall differences were observed by the Kruskal–Wallis test; however, * DSCF post hoc pairwise comparisons did not identify significant differences between specific groups (*p* > 0.05)

## Discussion

The present study evaluated patient-reported orofacial and masticatory symptoms before and after BoNT-A application in individuals presenting bruxism-related complaints through an online questionnaire. A statistically significant reduction in total symptom scores was observed after treatment, with more than a 52% decrease in self-reported pain and functional limitation. Overall, participants reported reduced masticatory discomfort and orofacial symptoms following BoNT-A application.

The results may be associated with the neuromuscular and antinociceptive effects of BoNT-A. Botulinum toxin type A acts primarily by inhibiting acetylcholine release at the neuromuscular junction, leading to temporary modulation of muscle contraction [10–12]. In addition to its local neuromuscular effects, growing evidence suggests that BoNT-A may also exert peripheral and central antinociceptive actions. Its analgesic effects have been associated with inhibition of neurotransmitter and neuropeptide release—including substance P, calcitonin gene-related peptide (CGRP), glutamate, and adenosine triphosphate (ATP)—through cleavage of the soluble N-ethylmaleimide-sensitive factor attachment protein receptor (SNARE) protein synaptosomal-associated protein of 25 kDa (SNAP-25) [10–12]. These processes may reduce peripheral sensitization and inflammatory signaling by limiting excitatory input from nociceptive afferents and decreasing activation of neurokinin-1 (NK-1) and glutamate receptors in dorsal horn neurons [12]. BoNT-A may also modulate central pain pathways via retrograde axonal transport and suppression of glial cell activation [11]. Together, these mechanisms may partially explain the reduction in patient-reported pain, chewing difficulty, and discomfort observed after treatment.

In agreement with the present findings, several studies have reported improvement in pain-related and muscle-related symptoms following BoNT-A application in patients presenting bruxism-related complaints or temporomandibular symptoms [1, 7, 12]. The symptomatic improvement observed in the present study suggests that BoNT-A may contribute to reduction of orofacial discomfort through neuromuscular and nociceptive modulation. Importantly, no participants reported worsening of chewing ability or additional discomfort after treatment, suggesting that the dose range used did not substantially impair masticatory function.

Sawadsopanont et al. (2025) [13] evaluated esthetic and functional outcomes of masseter BoNT-A injection in healthy adults and reported significant reductions in lower-face volume and improved satisfaction without changes in chewing function. Although their participants had no baseline dysfunction, their findings are consistent with the present study, in which no substantial functional impairment was reported following toxin application. Together, these findings suggest that BoNT-A may modulate muscle activity without substantially compromising everyday masticatory function. While Sawadsopanont et al. focused primarily on esthetic outcomes in healthy individuals, the present study evaluated symptom perception in patients presenting muscular and functional complaints. The improvement observed across different demographic groups suggests that the patient-reported benefits of BoNT-A may not be strongly dependent on sex-related anatomical differences or facial muscle characteristics.

Previous clinical and experimental studies have also reported reductions in electromyographic activity and patient-reported parafunctional symptoms following BoNT-A injections into the masseter and temporalis muscles. Serrera-Figallo et al. (2020) [5] and Shehri et al. (2022) [14] observed decreased muscle activity and reduction in bruxism-related manifestations after treatment. The questionnaire-based findings of the present study are consistent with these reports from a patient-reported perspective. Although the present study did not include objective physiological measurements, the observed symptom reduction supports the possibility that BoNT-A may contribute to improvement in muscle-related and orofacial symptoms in some clinical contexts.

Recent evidence suggests that BoNT-A injections for the management of masticatory myofascial pain and related symptoms are generally safe and well tolerated. According to the systematic review by De la Torre Canales et al. (2019) [15], the most commonly reported adverse effects include temporary regional weakness, tenderness at injection sites, and mild discomfort during chewing. Occasional reports of asymmetric smile, transient muscle reduction, and mild speech or swallowing difficulties have also been described. In the present study, no participant reported substantial worsening of chewing ability or functional limitation after treatment, suggesting that the injection protocol used was clinically well tolerated. Moreover, gradual reinnervation of motor end plates over time may contribute to recovery of muscle function following temporary neuromuscular modulation.

The use of an online, self-administered questionnaire facilitated data collection from a broad local population while maintaining confidentiality and adherence to CONEP (2021) ethical guidelines for virtual research. Patient-reported outcome measures are particularly valuable in studies involving orofacial pain and symptom perception because symptom intensity and impact on daily life are inherently subjective. Although this approach limits the ability to obtain objective physiological measurements, it provides an important perspective regarding patient perception and satisfaction following treatment [16]. The questionnaire design, which included identical items before and after BoNT-A application, enabled within-subject comparison and facilitated quantification of symptom changes over time.

Although a simplified questionnaire was used in the present study, it covered important dimensions commonly associated with orofacial pain and masticatory discomfort, including pain perception, functional limitation, and psychosocial impact. According to Villa et al. (2021) [7], health-related quality of life encompasses physical, psychological, and social domains that may be influenced by orofacial symptoms. Similar to previously described instruments such as Oral Health Impact Profile (OHIP) and Temporomandibular Joint Quality of Life (TMJ-QoL), the questionnaire used in the present study emphasized patient-reported functional and psychosocial outcomes. Therefore, the reduction in post-treatment scores may suggest improvement in aspects of daily life perceived by the participants.

Furthermore, exploratory analyses were performed to investigate whether demographic and occupational variables influenced symptom improvement after BoNT-A treatment. No significant association was observed between age and symptom improvement, and no significant difference was found between women and men. Although an overall difference in symptom improvement was detected among professional groups, post hoc pairwise comparisons did not identify significant differences between specific occupational categories. Previous studies have reported possible associations between age, sex, occupational stress, and variations in symptom perception among individuals with temporomandibular and bruxism-related complaints [17–20]. However, these associations were not confirmed in the present study, except for an overall difference among professional groups that should be interpreted with caution, as no significant pairwise differences were identified after post hoc analysis and some occupational categories included a limited number of participants.

Several limitations must be acknowledged. First, data were based solely on self-reported perceptions, without clinical or electromyographic confirmation. Although subjective assessments are valid for measuring quality-of-life outcomes, they may be influenced by personal bias or recall error. Second, the study lacked a control or placebo group, preventing definitive causal inference. The sample was non-probabilistic and regionally concentrated, which may limit external validity. The time interval between BoNT-A application and questionnaire completion was not standardized, as all eligible patients were invited regardless of the time elapsed since treatment. quently, participants may have completed the questionnaire at different stages of the therapeutic effect, introducing potential recall bias and variability in symptom reporting. Therefore, the findings should be interpreted as reflecting patients’ retrospective perceptions rather than outcomes assessed at a standardized follow-up time point.

Future investigations should include randomized controlled trials with larger and more diverse samples, longer follow-up periods, and integration of objective measures such as electromyography, bite-force analysis, or 3D facial scanning. Combining subjective and objective parameters would provide a more comprehensive understanding of how BoNT-A modulates both perception and performance of masticatory function. Additionally, studies comparing different dosages, injection sites, and intervals could optimize treatment protocols for various clinical scenarios.

## Conclusions

Individuals presenting bruxism-related complaints who received botulinum toxin type A (BoNT-A) application reported improvement in pain-related and masticatory symptoms from the patient’s perspective. The findings suggest that BoNT-A may represent a supportive approach for the management of orofacial and muscle-related complaints in selected clinical contexts. However, future studies using standardized diagnostic criteria, control groups, and objective outcome measures are necessary to better clarify the clinical effects and long-term outcomes associated with this approach.

## Data Availability

No datasets were generated or analysed during the current study.
